# A feasibility study of controlled human infection with *Streptococcus pneumoniae* in Malawi

**DOI:** 10.1016/j.ebiom.2021.103579

**Published:** 2021-09-24

**Authors:** Ben Morton, Sarah Burr, Tarsizio Chikaonda, Edna Nsomba, Lucinda Manda-Taylor, Marc Y.R. Henrion, Ndaziona Peter Banda, Jamie Rylance, Daniela M. Ferreira, Kondwani Jambo, Stephen B. Gordon, MARVELS consortium

**Affiliations:** a Malawi-Liverpool Wellcome Trust Clinical Research Programme, Queen Elizabeth Central Hospital, College of Medicine, P.O. Box 30096, Chichiri, Blantyre, Malawi; b Liverpool School of Tropical Medicine, Pembroke Place, Liverpool L3 5QA, United Kingdom; c Liverpool University Hospitals NHS Foundation Trust Liverpool L9 7AL, United Kingdom; d Queen Elizabeth Central Hospital, P.O. Box 95, Blantyre, Malawi; e College of Medicine, Private Bag 360, Chichiri, Blantyre, Malawi

**Keywords:** *Streptococcus pneumoniae*, Human infection model, Nasal, Mucosal inflammation

## Abstract

**Background:**

Persistent carriage of pneumococcal vaccine serotypes has occurred after introduction of PCV13 vaccination in Africa but the mechanisms are unclear. We tested the feasibility of using a human pneumococcal challenge model in Malawi to understand immune correlates of protection against carriage and to trial alternative vaccine candidates. We aimed to identify a dose of *Streptococcus pneumoniae* serotype 6B sufficient to establish nasopharyngeal carriage in 40% of those nasally inoculated and evaluate nasal mucosal immunity before and after experimental inoculation.

**Methods:**

Healthy student volunteers were recruited and inoculated with saline, 20,000 CFU/naris or 80,000 CFU/naris of *Streptococcus pneumoniae* serotype 6B Post inoculation carriage was determined by nasal sampling for bacterial culture and *lytA* PCR. Immunological responses were measured in serum and nasal mucosal biopsies before and after bacterial inoculation.

**Findings:**

Twenty-four subjects completed the feasibility protocol with minimal side effects. pneumococcal carriage was established in 0/6, 3/9 and 4/9 subjects in the saline, 20,000 CFU/naris and 80,000 CFU/naris groups, respectively. Incidental (natural) serotype carriage was common (7/24 participants carried non-6B strains, 29.2%. Experimentally induced type 6B pneumococcal carriage was associated with pro-inflammatory nasal mucosal responses prior to inoculation and altered mucosal recruitment of immune cells post bacterial challenge. There was no association with serum anti-capsular antibody.

**Interpretation:**

The serotype 6B experimental human pneumococcal carriage model is feasible in Malawi and can now be used to determine the immunological correlates of protection against carriage and vaccine efficacy in this population.

**Research in context**

**Evidence before this study**

Experimental human pneumococcal carriage has been used to measure humoral and cellular responses in mucosal and systemic compartments and as a method to test new vaccines in high income settings. However, the greatest burden of invasive pneumococcal disease is found in low- and middle- income countries, particularly in sub-Saharan Africa. Despite the roll out of pneumococcal conjugate vaccines in Malawi, vaccine serotype carriage remains high, leading to persistent population transmission and constraining herd effects. This is particularly problematic in the context of prevalent pneumococcal disease vulnerable populations (e.g., people living with HIV and adults exposed to excessive household air pollution and malnourishment) who are not eligible for vaccination under the WHO expanded programme on immunisation. There is currently poor understanding of the correlates of pneumococcal immunity and vaccine efficacy in these settings. Our group has conducted thirteen individual clinical studies with more than 1500 participants recruited in Liverpool, United Kingdom. A placebo-controlled trial of PCV13 demonstrated a 78% reduced carriage acquisition after nasal challenge in the United Kingdom. Multiple studies have demonstrated the safety of this predominantly asymptomatic colonisation model with mild symptoms of rhinorrhoea, sore throat, earache, fever and malaise infrequently reported.

**Added value of this study**

We report the establishment of a controlled human infection model of *Streptococcus pneumoniae* in Malawi, a low-resourced setting with high burden of invasive pneumococcal disease. This model and associated standard operating procedures were transferred from the United Kingdom. We showed that Malawian adult healthy volunteers can be safely challenged with serotype 6B pneumococcus, and that colonisation can be confirmed through classical microbiological culture of nasal wash. These observations were supported by findings of microbiological and immunological investigations

**Implications of all the available evidence**

Pneumococcal epidemiology in sub-Saharan Africa is characterised by higher rates of carriage and transmission than HIC settings despite introduction of conjugate vaccines. We have safely transferred this *S. pneumoniae* controlled human infection model to Malawi and this is now ready to be used as a platform to understand host-pathogen interactions, evaluate immune responses to current conjugate vaccines and test new vaccine candidates.

Alt-text: Unlabelled box

## 1 Introduction

Even in the era of conjugate vaccines, 400,000 deaths in children under 5 years were attributed to *Streptococcus pneumoniae* infection in 2015 [1]. African countries were disproportionately impacted with an estimated 2.4 million cases of invasive disease and 170,000 pneumococcal-attributable deaths in this period [1]. There is a persistent high burden of pneumococcal disease in both adults and children in Malawi which was associated with high residual vaccine-serotype (VT) pneumococcal carriage in children already vaccinated with 13-valent pneumococcal conjugate vaccine (PCV-13) [2]. The immunological mechanism to explain high VT pneumococcal carriage prevalence is not understood, but high population density and infection pressure are likely to be important.

For ten years, we have used an experimental human pneumococcal carriage model in the United Kingdom to study mucosal immunity against pneumococcal carriage and to test vaccine efficacy [3], [4], [5]. This model has demonstrated that vaccination with PCV13 reduced subsequent pneumococcal carriage acquisition by 78% in a UK population [3] and has provided important insights into the interactions between pneumococcal and influenza infection [6]. From an immunological perspective, the model has advanced our understanding of pneumococcal disease pathogenesis including the critical role of polysaccharide-specific memory B cells [7], nasal innate immunity [8] and alveolar macrophages [9].

Given the knowledge gaps related to VT pneumococcal carriage in high burden countries, we conducted a feasibility study to safely establish experimental pneumococcal carriage in Malawi and used novel nasal microbiopsy techniques to evaluate mucosal immunity before and after experimental inoculation. This work aims to evaluate which vaccines can prevent acquisition of pneumococcal carriage in Malawi, and to determine the critical mechanisms of respiratory tract defence that may be harnessed to develop more effective vaccines suitable for low- and middle-income country use.

## 2 Methods

### 2.1 Study protocol and randomisation

This study adhered to a published protocol (https://wellcomeopenresearch.org/articles/5–25)^10^. Briefly, healthy participants aged 18–40 years consented to nasal inoculation of *Streptococcus pneumoniae* serotype 6B to determine the proportion who subsequently developed colonisation. The majority of participants were undergraduate students from adjacent university campuses. Fluency in written Chichewa or English (to follow safety instructions) were inclusion criteria but no other sampling frame restrictions were imposed [10]. A detailed medical history and clinical examination was performed during screening. Exclusion criteria included HIV seropositivity, pregnancy and colonisation with pneumococcal serotype 6B (other serotypes permitted) at the screening visit.

We tested two doses of inoculum (20,000 and 80,000 colony forming units per naris) in blocks of 12 participants, each block randomised 3:1 with 9 participants receiving pneumococci and 3 receiving saline (0.9%). Rather than using a formal power calculation for this feasibility study, we hypothesised that the infection rate in Malawi would be similar to that in the UK and therefore that a dose of 80,000 colony forming units per naris would be sufficient to establish nasal carriage in 40–50% of participants [3]. We therefore defined feasibility *a priori* as four or more participants in either dose escalation group developing experimental carriage. This feasibility study transferred an established technique where more than 1500 participants have been inoculated with pneumococci with well-established carriage rates [10]. A secondary objective was to establish the feasibility of immunological and diagnostic standardised operating procedures transferred from our UK model to Malawi.

### 2.2 Ethical approvals

The study was ethically approved in Malawi by the National Health Sciences Research Committee (NHSRC, reference number: 19/08/2246) and in the United Kingdom by the Liverpool School of Tropical Medicine (LSTM, reference number: 19-017). LSTM also sponsored the study. Informed written consent was recorded for all participants. Deidentified participant data will be made openly available one year after the publication date using the Harvard Dataverse platform

### 2.3 Symptoms, sampling and endpoints

We determined participant symptoms including expected adverse events with open and targeted questions at every clinic visit. Intermittent sampling was performed for immunological analysis at 5 days before intranasal pneumococcal inoculation, and then on days 2, 7, and 14 afterwards: at these time points we collected nasal wash for bacterial detection, peripheral blood, nasal scrapes and nasosorption samples. The primary endpoint was detection of the inoculated pneumococci serotype (6B) by classical culture from nasal wash recovered from the participants at any of days 2, 7 and 14 after pneumococcal challenge. Nasal wash is established as the diagnostic technique from the Liverpool model due to the theoretical risk of nasopharyngeal trauma and pneumococcal invasion from nasal swabbing. In parallel, we confirmed carriage by detection of pneumococcal *lyt*A by PCR on nasal wash samples. Secondary endpoints were symptoms, mucosal and systemic immune parameters. All subjects completed exit questionnaires to assess study experience.

### 2.4 Preparation, inoculation and culture detection of pneumococcus

Briefly, as described in our study protocol [10], an aliquot of *S. pneumoniae* serotype 6B BHN418 was thawed, centrifuged and washed before being resuspended in 0.9% normal saline at the pre-specified concentration (20,000 CFU/100 µl or 80,000 CFU/100 µl). The prepared inoculum was taken immediately (less than 5 min transit) to the clinical area where 100 µl was instilled into each nostril of the participant [3].

Nasal wash was collected as described elsewhere [3]. Briefly, 5 ml of 0.9% saline was instilled into each naris; this was repeated twice (10 ml per naris, 20 ml total). Samples were transported to the laboratory and centrifuged at 3400 g for 10 min. Following centrifugation, 1 ml aliquots of the supernatant were stored at −80 °C. The nasal wash bacterial pellet was resuspended in 100 µl of skim milk, tryptone, glucose, and glycerine (STGG). Samples were retained for bacterial culture and DNA extraction followed by *lytA* PCR.

To quantify colonization density by culture, serial dilutions of the pellet, in 100 µl STGG, were plated on Columbia sheep Blood Agar (Oxoid, UK) containing 4 mg/ml gentamicin (CBG). Plates were incubated at 37 °C with 5% CO_2_ for 18–24 h and inspected to identify pneumococcal phenotype. Serotype (23-valent kit) was confirmed by latex agglutination using Immulex Pneumotest reagents (Statens serum institute).

### 2.5 Pneumococcal lytA PCR analysis

Genomic bacterial DNA was isolated from nasal wash pellets stored in STGG using the Agowa Mag mini-DNA extraction kit (LGC Genomics, Berlin, Germany) as per manufacturer's instruction using a 300 µl aliquot of the stored nasal wash STGG pellet (the original 100 µl preparation was resuspended into 800 µl STGG and stored in three aliquots). The eluted DNA was stored at −20 °C for use in *lytA* qPCR. PCR amplification of the *lytA* gene was carried out using forward primer (5′-ACGCAATCTAGCAGATGAAGCA-3′) and reverse primer (5′-TCGTGCGTTTTAATTCCAGCT-3′). Master mix was prepared using 12 µl of DEPC-treated H_2_O; 0.225 µl of each primer (100 µM); 0.125 µl of probe (100 µM); 12.5 µl of Taq. The total volume of PCR reaction used was 25 µl, containing: 22.5 µl of the prepared master mix and 2.5 µl of sample in a set of duplicate wells. Finally, 2.5 µl of *lytA* plasmid (Fast-Track Diagnostics, Luxembourg) making up the standard curve was added into each of two wells, beginning with the lowest concentration standard (1 × 10° copies/µl) to the highest concentration (1 × 10^5^ copies/µl). The plate was loaded in a thermocycler (QuantStudio 7 Flex, Applied Bioscience) with the following amplification conditions: 95 °C for 10 min; followed by 40 cycles of 95 °C for 15 s and then 60 °C for 1 min.

### 2.6 Nasal biopsy and flow cytometric mucosal immunophenotyping

Nasal microbiopsies were obtained from the inferior turbinate of each participant using microcurettes without local analgesia. Each sample was dropped immediately into transport medium and transferred to the laboratory in less than 10 min. For immunophenotyping, nasal cells were dislodged from curettes by gentle pipetting and suspension. Cells were then live-stained with an antibody cocktail containing anti-human CD3 APC, anti-human CD19 Brilliant Violet 510, TCR Gamma Delta FITC and anti-human CD45 Alexa Fluor 700 (all BioLegend), anti-human CD14 PE-Cy7 (BD Biosciences), anti-human CD66b PE (eBiosciences) and MR1 Tetramer Bv421 (NIH Tetramer Core Facility). After fixation, samples were acquired using standard methods on an LSR FORTESSA flow cytometer (BD Biosciences) and analysed using Flowjo v10.5.3 (BD Biosciences).

### 2.7 Nasal lining fluid collection and luminex analysis of nasal fluid cytokines

Nasosorption filters were gently applied to the nasal mucosa, transferred in dry collection tubes and stored at −80 °C. Cytokines were eluted from stored nasosorption filters (Mucosal Diagnostics, Hunt Developments (UK) Ltd., Midhurst, UK) using 200  μl of elution buffer (Millipore) by centrifugation at 1500 g, then the eluate was cleared by further centrifugation at 1595 g. The samples were acquired on a MAGPIX (Luminex, UK) using a 38-plex magnetic human cytokine kit (Millipore) and analysed with xPONENT software following the manufacturer's instructions. The analytes included sCD40L, EGF, Eotaxin/CCL11, FGF-2, Flt-3 ligand, Fractalkine, G-CSF, GM-CSF, GRO, IFN-α2, IFN-γ, IL-1α, IL-1β, IL-1ra, IL-2, IL-3, IL-4, IL-5, IL-6, IL-7, IL-8, IL-9, IL-10, IL-12 (p40), IL-12 (p70), IL-13, IL-15, IL-17A, IP-10, MCP-1, MCP-3, MDC (CCL22), MIP-1α, MIP-1β, TGF-α, TNF-α, TNF-β and VEGF.

### 2.8 Anti-pneumococcal capsular polysaccharide (PS) ELISA

Anti-pneumococcal capsular PS antibodies were determined in serum using the World Health Organization internationally standardized method and reagents [7]. Briefly, 96-well ELISA plates were coated using 5 μg/mL of purified serotype 6B polysaccharide (Statens Serum Institute) overnight at 4 °C. Plates were washed 3 times with PBS containing 0.05% Tween-20 between each step. Wells were blocked with 1% Bovine Serum Albumin in PBS for 2 h at room temperature. Samples were diluted in PBS containing 5 μg/mL CWPS Multi (Statens Serum Institute) and incubated for 30 min at 37 °C. 007sp reference serum received from NIBSC was used as a standard. Diluted/adsorbed samples were then transferred to pre-coated plates and incubated for 2 h at room temperature. Antibody detection was performed using IgG-Horseradish Peroxidase and TMB substrate-based platform. All samples were run in triplicate in four dilutions, and samples with a CV of greater than 15% were repeated. Results are expressed as μg/mL calculated using the assigned IgG concentrations in reference serum 007sp.

### 2.9 Statistical analyses

Statistical analyses were performed using R v.4.0.2 (R Development Core Team, Vienna, Austria) and GraphPad Prism v9.0.0 (GraphPad Software, San Diego, California, USA). Non-normally distributed quantitative measurements are summarised by the median and inter-quartile range (IQR). Exact binomial confidence intervals are reported for estimated proportions. Categorical variables were compared using Fisher's exact test. The nasal cell and serum IgG antibody data were analysed using Friedman test with correction for multiple comparisons using Dunn's multiple comparisons test. A *p*-value of < 0.05 was considered statistically significant, all *p*-values are two-tailed. All cytokine data were log_10_-transformed and summarised by medians and IQRs.

### 2.10 Role of funding source

The funder and sponsor have no role in study design; collection, management, analysis, and interpretation of data; writing of the report; or the decision to submit the report for publication.

## 3 Results

### 3.1 Recruitment, inoculation and symptoms

**Fig. 1:**
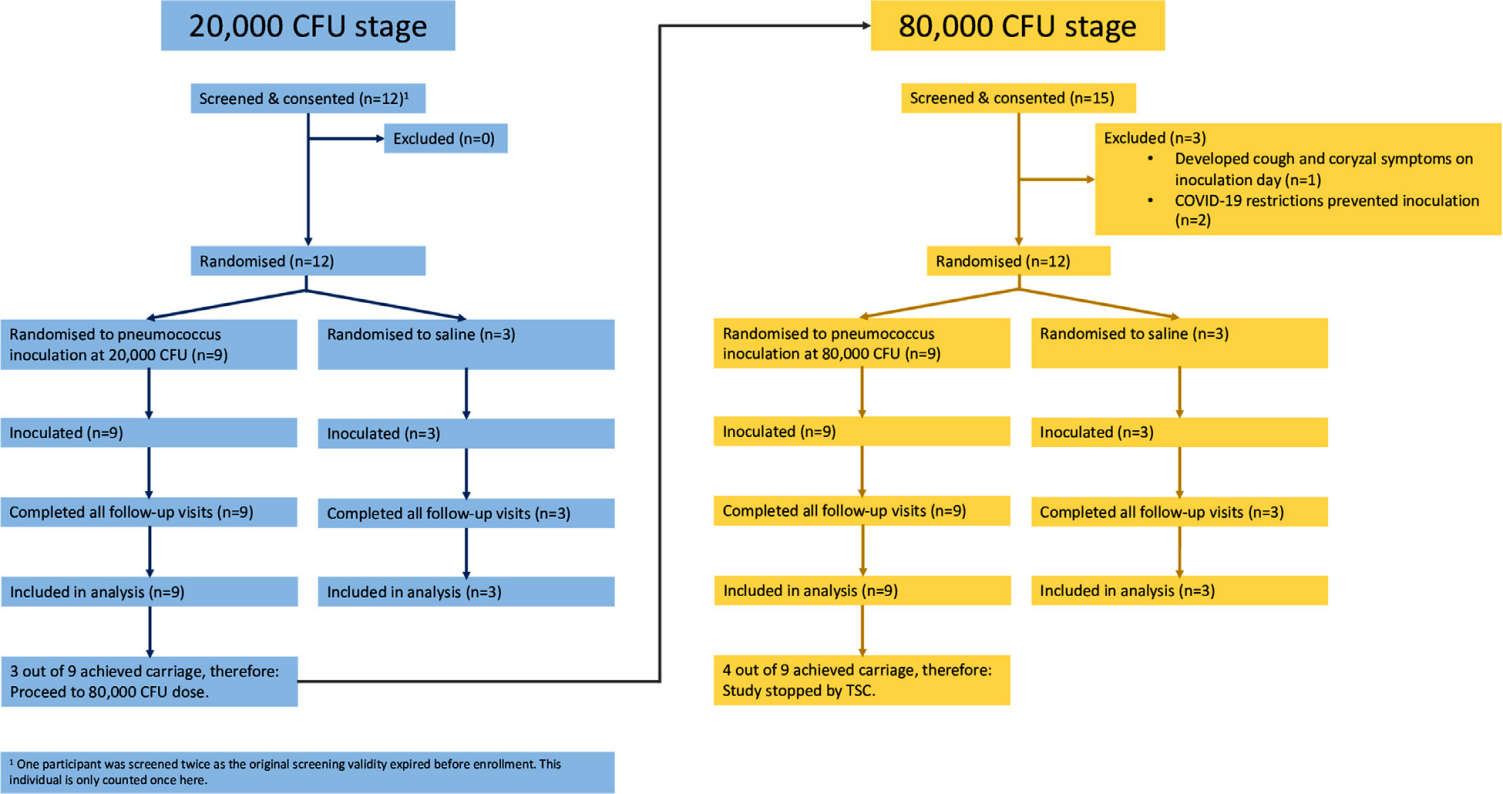
**CONSORT diagram.** Figure demonstrates the number of participants screened and subsequently inoculated within the study. An additional two participants were screened for the planned 160,000 CFU group but the study met pre-defined stopping criteria before inoculation [10].

Twenty-nine participants were screened and 24 inoculated (Fig. 1) before the study met *a priori* defined stopping criteria [10]. Twelve participants were randomised (3:1) to receive inoculation with *S. pneumoniae* serotype 6B strain BHN418 20,000 colony forming units (cfu)/naris or normal saline (i.e. *n* = 9 randomised to *S. pneumoniae* serotype 6B and *n* = 3 to normal saline) and 12 subjects were randomised (also 3:1) to the higher inoculum of 80,000 cfu/naris or normal saline (again *n* = 9 for *S. pneumoniae* and *n* = 3 for saline). All inoculated participants completed the study follow up as per protocol, and all reported a satisfactory experience. The study commenced November 2019 and was paused (at *n* = 20 recruited participants) due to the emerging COVID-19 pandemic in March 2020. The study recommenced in October 2020 with additional safety procedures (including COVID-19 testing and the provision of additional personal protective equipment for research staff). Participant characteristics are described in Table 1. Following inoculation, minor symptoms were observed in 9/24 of inoculated participants, consistent with previously published work [12]. Whilst not statistically significant, we noted that 4/6 participants (66.7%) inoculated with saline developed symptoms, compared to 5/18 participants (27.8%) inoculated with pneumococcus (Table 2). We also noted that there were more participants who smoked 2/9 and drank alcohol 6/9 in the 80,000 CFU group (Table 1). These observations require follow up in future studies.

**Table 1:** Participant demographics. BMI: body mass index.

	All (*n* = 24)	Control (*n* = 6)	20,000 CFU (*n* = 9)	80,000 CFU (*n* = 9)
Age [median (IQR)]	23 (22,25)	24 (23,26)	23 (20,25)	23 (22,24)
Sex [male (%)]	20 (83.3)	5 (83.3)	7 (77.8)	8 (88.9)
Current smoker [n (%)]	2 (8.3)	0 (0.0)	0 (0.0)	2 (22.2)
Occasional alcohol [n (%)]	9 (37.5)	0 (0.0)	3 (33.3)	6 (66.7)
BMI [median (IQR)]	22.9 (20.3,24.2)	23.3 (19.0,24.0)	22.7 (19.9,23.1)	23.1 (21.1,26.6)

Both current smokers both smoked less than five cigarettes per day. Twenty participants had never smoked, and two participants had given up smoking more than six months previously with less than 10 pack years.

**Table 2:** **Participant symptoms after inoculation.** Participants were specifically asked if they had experienced listed symptoms at each follow up visit after inoculation. “Other” symptoms were: one episode of vomiting thought to be related to food, one episode of non-specific tiredness and one episode of “wet nose at night-time”. Symptoms reported within the table represent aggregated data from participants during symptom screening two, seven and fourteen days after inoculation. Participants classified as colonised with 6B if this serotype was detected in any nasal wash sample two, seven and fourteen days after inoculation.

	*n*	Total AEs	Sore throat	Coryzal symptoms	Headache	Earache	Feverish	Cough	Rash	Other
All	24	9	0	3	2	0	0	0	1	3
Control	6	4	0	2	2	0	0	0	0	0
20,000 CFU	9	4	0	1	0	0	0	0	0	3
80,000 CFU	9	1	0	0	0	0	0	0	1	0
Colonised (6B)	7	2	0	1	0	0	0	0	0	1
Colonised (not 6B)	6	2	0	0	0	0	0	0	1	1
Not colonised	11	5	0	2	2	0	0	0	0	1

### 3.2 Primary endpoint pneumococcal carriage and incidental non-experimental carriage

**Fig. 2:**
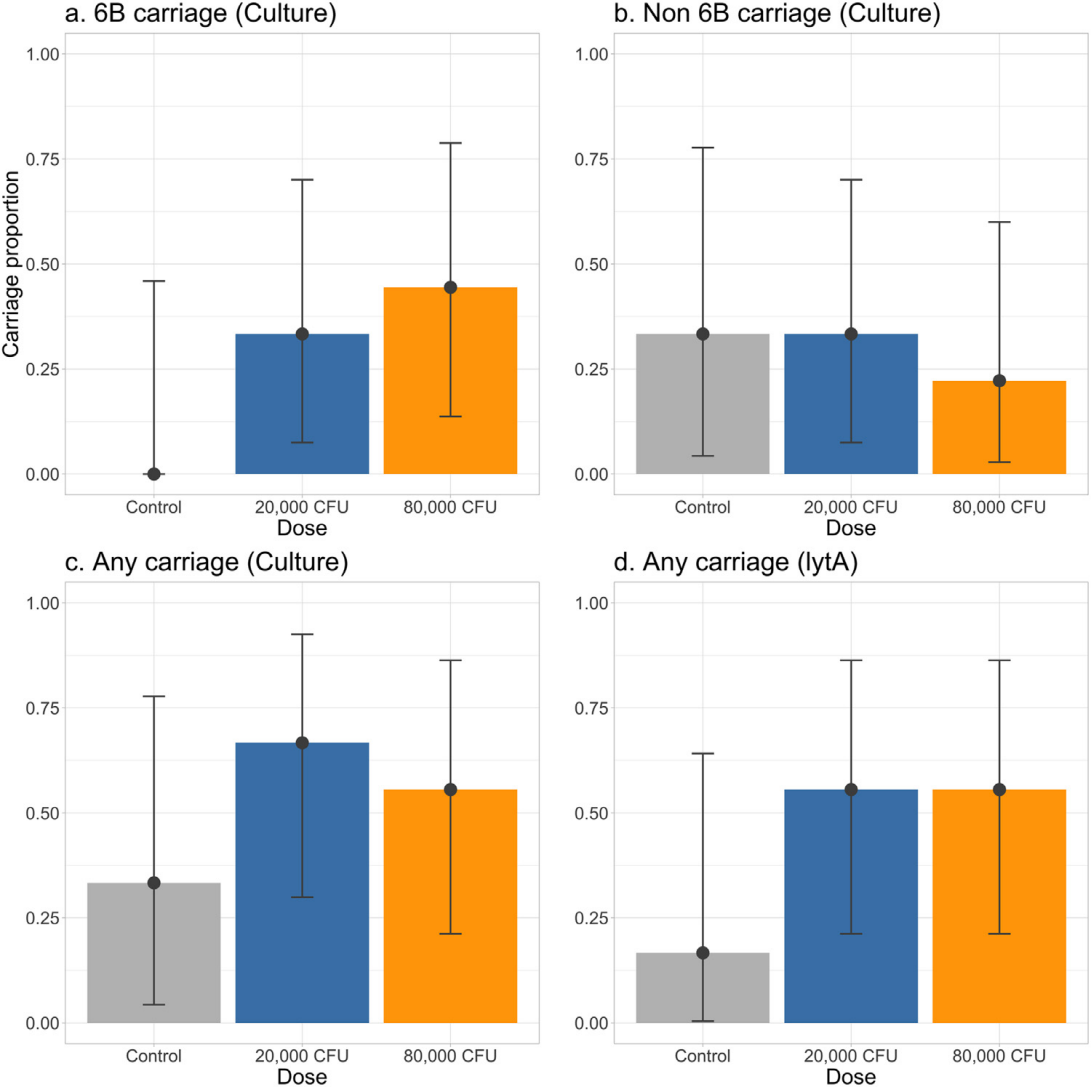
**Pneumococcal detection from nasal wash.** Figure demonstrates the proportions (with confidence intervals) of pneumococcal serotype 6B (panel a) and non-6B carriage (panel b) in participants from the control, 20,000 and 80,000 CFU/naris groups. Presence of any pneumococcal nasal carriage was determined by both culture and *lytA* PCR as shown in panels c and d.

Inoculation with pneumococcal serotype 6B, resulted in 3/9 (33% [CI 7–70]) and 4/9 (44% [CI 14–79]) of participants developing nasal colonisation, in the 20,000 CFU and 80,000 CFU groups, respectively. The inoculation doses (median (IQR) of serotype 6B were 21,834 (19,500–22,333) and 91,667 (88,334–100,000) for the 20,000 and 80,000 CFU groups, respectively. The prevalence of incidental carriage was relatively common with non-6B serotypes identified in 7/24 (29.2% [13–51]) participants (Fig. 2) These included both vaccine serotypes (7, 17–19) and non-vaccine serotypes (found in 9 samples from 3 participants). One of these participants demonstrated serotype 18 carriage during screening and was subsequently colonised with 6B after nasal challenge. A full description of nasal wash results including culture (CFU/100 µl) and *lytA* density (copies per ml) is provided in the supplementary material (Table S1). We found high concordance between classic microbiological culture and *lytA* PCR positivity for *S. pneumoniae* (84 out of 96 concordant samples, 87.5%). Serotype 6B specific concordance could not be calculated as *lytA* detection cannot distinguish different serotypes.

### 3.3 Cellular infiltration of the nasal mucosa following pneumococcal challenge

**Fig. 3:**
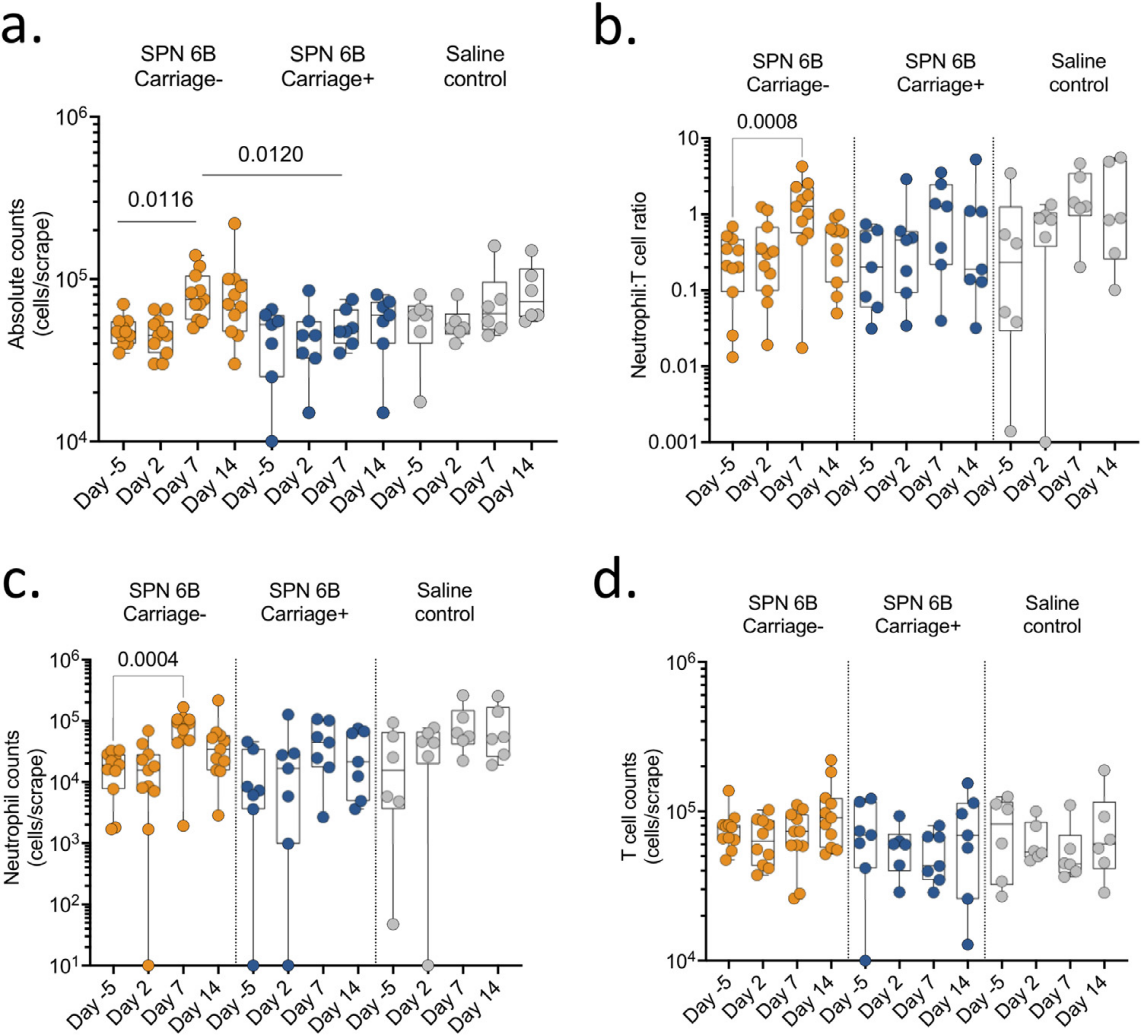
**Nasal cellular immune responses before and following inoculation.** Nasal cells were collected by mucosal scraping with rhinoprobes. A cell scrape from each nostril were combined for flow cytometry-based immunophenotyping. (a) Total absolute cell counts from the cell scrapes at different time points showed increased cells at day 7 in the group that did not develop carriage with uniform collections at other points. (b) Neutrophil to T cell ratio from the flow cytometry based immunophenotyping shows increase in carriage negative group at day 7. (c) Absolute neutrophil counts from the cell scrapes at different time points by experimental condition. (d) Absolute T cell counts from the cell scrapes at different time points by experimental condition showing no difference. The horizontal bars represent the median and interquartile range (IQR). Data were analysed using Friedman test with correction for multiple test using Dunn's multiple comparisons test, comparing baseline to day 2, 7 and 14 within each study group (Carriage positive, *n* = 7; Carriage negative, *n* = 11; Saline controls, *n* = 6). SPN, *Streptococcus pneumoniae*.

Immune responses induced by respiratory pathogens are generally compartmentalised to the respiratory mucosa [13,14]. We therefore sought to explore whether nasal pneumococcal challenge or carriage induced cellular responses in the nasal mucosa, and if they are associated with development of pneumococcal carriage. We measured the changes in numbers and proportions of immune cells collected using nasal curettes from the nasal mucosa. Nasal scrapes collected per individual for immunophenotyping resulted in a cell yield of 27,500 cells/scrape [IQR 22,500-37,500]. The number of immune cells increased between baseline and day 7 post pneumococcal challenge in carriage negative individuals (*p* = 0.012, Friedman test with Dunn correction), but this was not observed in the experimental carriage positive individuals or those that received normal saline (Fig. 3a). Moreover, the number of immune cells was higher at day 7 in the carriage negative individuals compared to the carriage positive individuals (*p* = 0.012, Kruskal-Wallis with Dunn's correction), but was similar with those that received normal saline (*p* = 0.533, Kruskal-Wallis with Dunn's correction) (Fig. 3a). Neutrophils and T cells were the predominant immune cells in nasal mucosal samples (Supplementary Fig. 1), hence we focused our analysis on these major immune cells. The neutrophil to T cell ratio increased between baseline and day 7 in all study groups, but the difference was only statistically significant in the in carriage negative individuals (*p* < 0.001, Friedman test with Dunn correction) (Fig. 3b–c), likely due to the small sample size. An analysis on the absolute numbers also showed an increase in numbers of neutrophils between baseline and day 7 in all the study groups, but this was also only statistically significant in the in carriage negative individuals (*p* = 0.004, Friedman test with Dunn correction) (Fig. 3d), suggesting that baseline sampling may have induced infiltration of neutrophils to the nasal mucosa. In contrast, no changes were observed in nasal T cell numbers between baseline and day 7 in all the study groups (Fig. 3d). In addition, there were no differences in the frequencies of B cells, GD T cells, MAIT cells and monocytes overtime and across the study groups (data not shown). Together, these results suggest that an attenuated cellular infiltration in the nasal mucosa may be associated with successful experimental introduction of pneumococcal serotype 6B carriage.

### 3.4 Association of nasal cytokine environment at inoculation and subsequent development of carriage

**Fig. 4:**
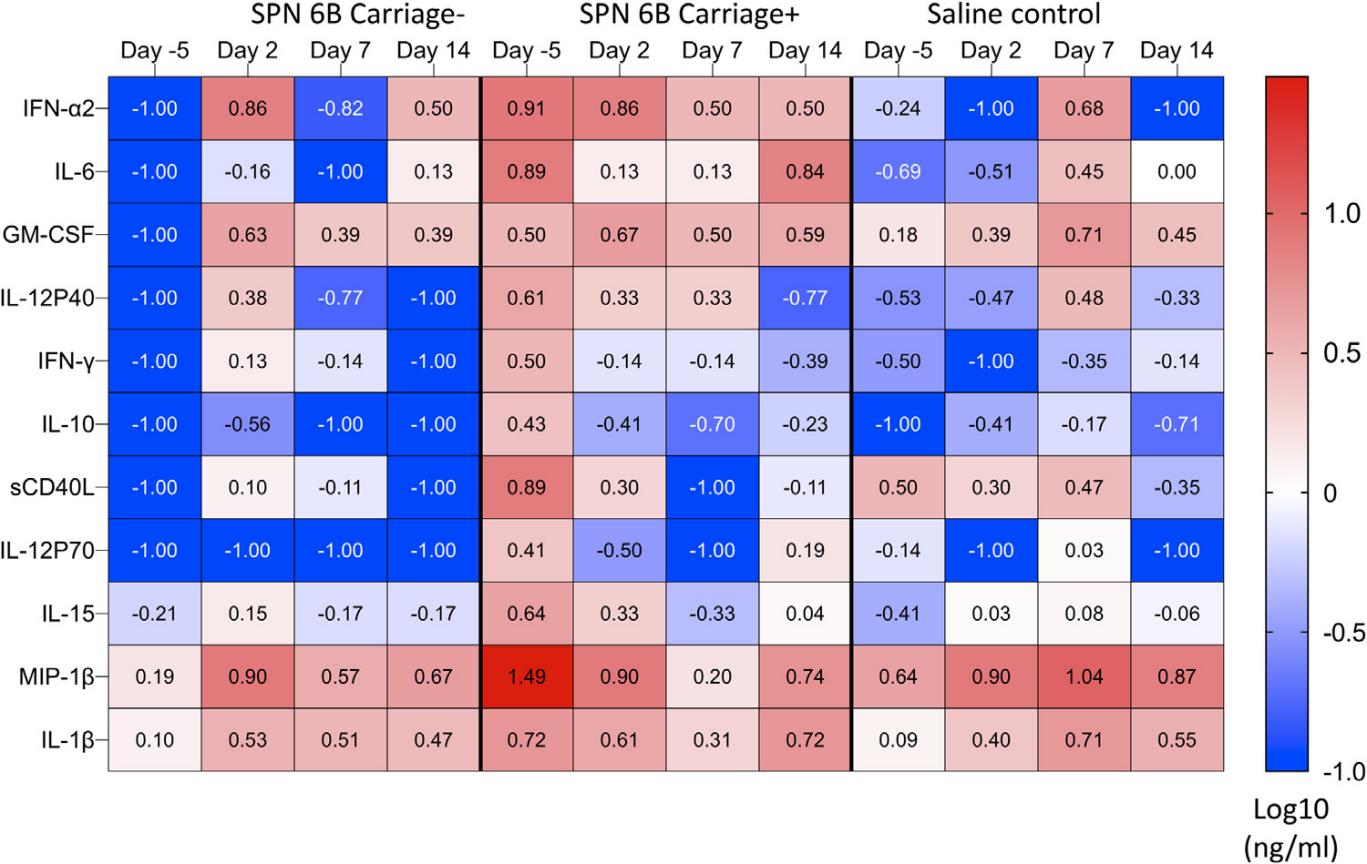
. **Nasal cytokine responses before and following inoculation.** Nasal lining fluid was collected using nasosorption filters. Nasal lining fluid was eluted from nasosorption filters and cytokines were measured using a 38-plex magnetic human cytokine kit. The data was log 10 transformed and plotted on a heatmap as median concentrations of selected cytokines in nasal lining fluid among the study groups, at multiple time points. Blue represents low concentration and red represents high concentrations. The data from day −5 in each panel show that the group developing carriage (middle panel) had higher median levels of inflammatory cytokines that the group who did not develop carriage (left hand panel). Saline controls were intermediate as expected. Carriage positive, *n* = 7; Carriage negative, *n* = 11; Saline controls, *n* = 6. SPN, *Streptococcus pneumoniae*. (For interpretation of the references to color in this figure legend, the reader is referred to the web version of this article).

Nasal inflammation has been associated with increased propensity for experimental pneumococcal carriage [8] and carriage in children in Kenya [15]. We therefore sought to explore whether the baseline cytokine environment before pneumococcal challenge influenced subsequent development of pneumococcal carriage in our model. Concentrations of 37 cytokines and sCD40L were measured in nasal lining fluid (Supplementary Fig. 2). Concentrations of Flt-3 L, IL-3 and TNF-β were not detectable in any of the samples. There was a trend towards higher levels of IFN-α2, IL-6, GM-CSF, IL12p40, IFN-γ, IL-10, sCD40L, IL-12p70, IL-15, MIP-1β and IL-1β at baseline (day −5), in those subjects that eventually successfully developed experimental pneumococcal carriage (carriage positive) compared to those that did not (carriage negative) or subjects given saline as controls (Fig. 4 and Supplementary Fig. 2). Further work in larger cohorts is required to elucidate cytokine changes that occur in response to inoculation. The findings do suggest that an inflammatory nasal cytokine microenvironment prior to inoculation may indeed be associated with successful development of serotype 6B carriage.

### 3.5 Nasal pneumococcal challenge or carriage did not elicit an increase in serum anti-6B polysaccharide igG antibodies

**Fig. 5:**
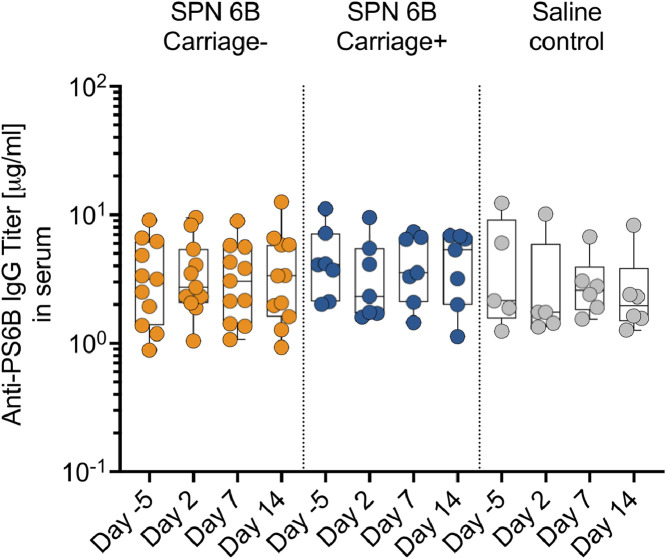
**Anti-6B polysaccharide IgG antibodies in serum before and following inoculation.** Levels of polysaccharide 6B (PS6B)-specific IgG antibodies in serum were measured using an enzyme-linked immunosorbert assay (ELISA). The serum samples were from volunteers experimentally inoculated with *Streptococcus pneumoniae* 6B, and those who received a normal saline as a control. The *S. pneumoniae* inoculated volunteers were further subdivided into carriage positive (carriage+) and carriage negative (carriage-). The serum samples were obtained before (Day −5) and after (Days 3, 7, and 14) inoculation. The horizontal bars represent the median and interquartile range (IQR). Data were analysed using Friedman test with correction for multiple test using Dunn's multiple comparisons test, comparing baseline to day 2, 7 and 14 within each study group (Carriage positive, *n* = 7; Carriage negative, *n* = 11; Saline controls, *n* = 6). There were no significant differences in IgG level in any experimental condition or at any time point. One individual from the Saline control group does not have a serum IgG result at Day −5 and Day 2. IgG, immunoglobulin G; SPN, *Streptococcus pneumoniae.*

Serum anti-6B polysaccharide IgG antibody levels have been shown to increase in subjects that establish pneumococcal carriage following nasal experimental challenge with serotype 6B [7]. We measured the levels of anti-6B polysaccharide IgG antibodies in serum before (day −5) and after (day 2, 7 and 14) inoculation with normal saline or pneumococcal serotype 6B There was no difference between groups at baseline and we did not observe an increase in the serum anti-6B polysaccharide IgG antibody levels in those that were inoculated compared to those that were not, irrespective of the serotype 6B carriage status (all *p* > 0.05, Friedman test with Dunn correction). These findings suggest that experimental nasal pneumococcal challenge or experimental human pneumococcal carriage did not induce serum anti-6B polysaccharide IgG levels beyond natural exposure in this setting (Fig. 5). Further work with larger numbers of subjects will be required to determine if small rises in IgG are induced by experimental carriage.

## 4 Discussion

This study has shown for the first time that experimental human pneumococcal carriage can be safely carried out in Malawi. Malawi has very different pneumococcal epidemiology and biology compared to Europe likely underpinned by differences in human-pathogen interaction as well as the physical environment, and socio-economic circumstances including the effects of crowding. Our primary objective was achieved in that our study showed that recruitment, screening of volunteers and safe completion of the study per protocol was feasible with local adaptation of the protocol initially developed in the UK. Observed carriage rates were consistent with that observed in the UK and the model behaved similarly. There were no specific local difficulties with recruitment, inoculum was delivered accurately and safely and the rates of experimental pneumococcal carriage mirrored those from our Europe-based studies.

Our secondary objectives were to measure carriage symptoms and to determine if immunological sampling would be possible in sufficient yield to study key differences in carriage epidemiology observed between the UK and Malawi [16]. As in Europe, we found experimental human pneumococcal carriage to be asymptomatic. Nasal fluid collection by nasosorption, nasal micro-biopsy and nasal wash were all well tolerated and full sample sets well collected from all subjects. As the sampling was successful, we were able to compare several immunological parameters between subjects who experienced experimental carriage and those who did not, as well as saline controls. These data showed evidence that experimental carriage following inoculation was associated with a specific baseline inflammatory milieu, and that the clearance of inoculum without establishment of carriage was associated with robust cellular infiltration of the nasal mucosa in day 7 (post-inoculation) samples. Further work is required in a larger sample to establish the relationship with cellular and cytokine responses. We did not find evidence that establishment of carriage was associated with baseline anti-capsular IgG nor did experimental carriage induce any increase in serum anti-capsular IgG.

This study in Malawi shows some similarity with work previously published from Liverpool, UK. The predominantly student population had no difficulty in completing the protocol [17]. The observed experimental pneumococcal carriage rate, albeit in small numbers so far, was similar to that observed in the UK [4]. The lack of specific symptoms for participants who developed experimental carriage were also as expected [5]. The observed association of inflammation with increased carriage rate was supportive of the observation in the UK that viral co-infection was associated with increased carriage [18]. This observation will be followed up with further studies of coincident asymptomatic viral infection in Malawi. The lack of association of experimental carriage with serum IgG was consistent with UK results [19] which showed that circulating B cells rather than serum IgG levels were associated with protection from carriage.

There were, however, some important differences in our study from the UK experience and from our expected results. The baseline and incidental naturally acquired pneumococcal carriage rate were higher than that in Liverpool, and so the usefulness of *lytA* PCR as a confirmatory method of determining carriage was much lower than in the UK. The frequency of naturally acquired carriage episodes in Malawi is therefore a factor that must be considered in experimental design using the human challenge model in future studies. Specifically, it may be important to use molecular methods to characterise the several serotypes present in the nasopharynx. It was surprising, however, that we saw no evidence of increased experimental carriage in those participants already experiencing natural carriage as our previous studies of the nasal microbiome suggested a “carriage tolerant” phenotype in participants with pre-existing pneumococcal colonisation [20]. This result will need to be further explored with larger numbers of subjects. It may also be useful to explore controlled human infection with pneumococcal serotypes that are more commonly found in Malawi.

The main strength of this study is that it shows experimental human pneumococcal carriage to be feasible in Africa, a continent with a residual burden of pneumococcal disease that may not be controlled by current conjugate vaccination strategy. A further strength of the study is that we piloted the use of sophisticated mucosal analyses to unpick immunological differences between Europe and Africa. This feasibility study has generated a number of hypotheses that should now be tested in larger cohorts, most important being the association of nasal mucosal inflammation with frequent pneumococcal carriage.

The main limitations in this study are related to the small numbers of participants. Larger studies will confirm that the rate of reported symptoms remain low. Further, we achieved accurate challenge dosing of controlled shipment from Liverpool [3,11]. It remains our goal to manage the entire production of the inoculum in Malawi in due course. The rate of infection will be confirmed in larger numbers of subjects as will the immunological observations in this cohort. We acknowledge that participant self-reporting or recall bias may be a potential limitation of this study, but participants (and clinical researchers) were blinded to inoculation allocation (saline vs. pneumococci) so this should not have influenced differentially observed reporting between these groups. In future studies, we will incorporate the use of multiplex lytA/6B PCR as lytA detection alone could not distinguish the different serotypes hence not being able to calculate serotype 6B specific concordance between culture and PCR. We have confirmed that the experimental human pneumococcal challenge model may now be used in Malawi to test novel vaccines, and to explore the differences that underpin the observed differences in epidemiology.

## Contributors

All authors read and approved the final version of the manuscript • Conceptualization BM, KJ, SB, EN, LMT, MYRH, NPB, JR, DMF, SBG • Data Curation and Verification BM, KJ, SB, TC, EN, LMT, MYRH, SBG • Funding Acquisition BM, KJ, JR, DMF, SBG • Investigation BM, KJ, SB, TC, EN, LMT, MYRH, NPB, JR, DMF, SBG • Methodology BM, KJ, SB, TC, EN, LMT, MYRH, NPB, JR, DMF, SBG • Project Administration BM, KJ, SB, EN, LMT, SBG • Resources BM, KJ, JR, DMF, SBG • Supervision BM, KJ, SB, TC, LMT, SBG • Validation BM, KJ, SB, EN, TC • Visualization BM, KJ, SB, TC, MYRH, SBG • Writing – Original Draft BM, KJ, SBG • Writing – Review & Editing BM, KJ, SB, TC, EN, LMT, MYRH, NPB, JR, DMF, SBG.

## Declaration of Competing Interest

The authors declare no competing interests.

## MARVELS consortium

• Clinical team: Clara Ngoliwa, Edward Mangani, Modesta Reuben, Vitumbiku Nkhoma and Helen Thomson
• Laboratory team: Christopher Mkandawire, Simon Sichone, Raphael Kamng'ona, Mphatso Mayuni, Percy Mwenechanya, Asia Sophia-Wolf, Godwin Tembo and Bridgette Galafa.
• Social Science: Neema Toto, Tina Harawa and Blessings Kapumba
• Data managers: Joel Gondwe and Clemens Masesa
• Administration: Sandra Antoine
• Collaborators: Kate Gooding, Markus Gmeiner, Mike Parker, Andrew Pollard, Rob Heyderman, Jason Hinds, Mark Alderson, Chris Bailey, Marien de Jonge, Robert Kneller, Jeremy Brown, Jane Mallewa, David Goldblatt, Richard Malley, Jeff Weiser, Jonathon Grigg, Henry Mwandumba, and Debby Bogaert

## Appendix B Supplementary materials

Image, application 1

Image, application 2

Image, application 3

Image, application 4

Image, application 5
