# Supplementary material for: A feasibility study of controlled human infection with Streptococcus pneumoniae in Malawi
Source: eBioMedicine. 2021 Sep 24;72:103579. doi: 10.1016/j.ebiom.2021.103579 (PMC8479630; doi:10.1016/j.ebiom.2021.103579)
Supplement: Supplementary file 1 [file mmc1.pptx]

## Slide 1
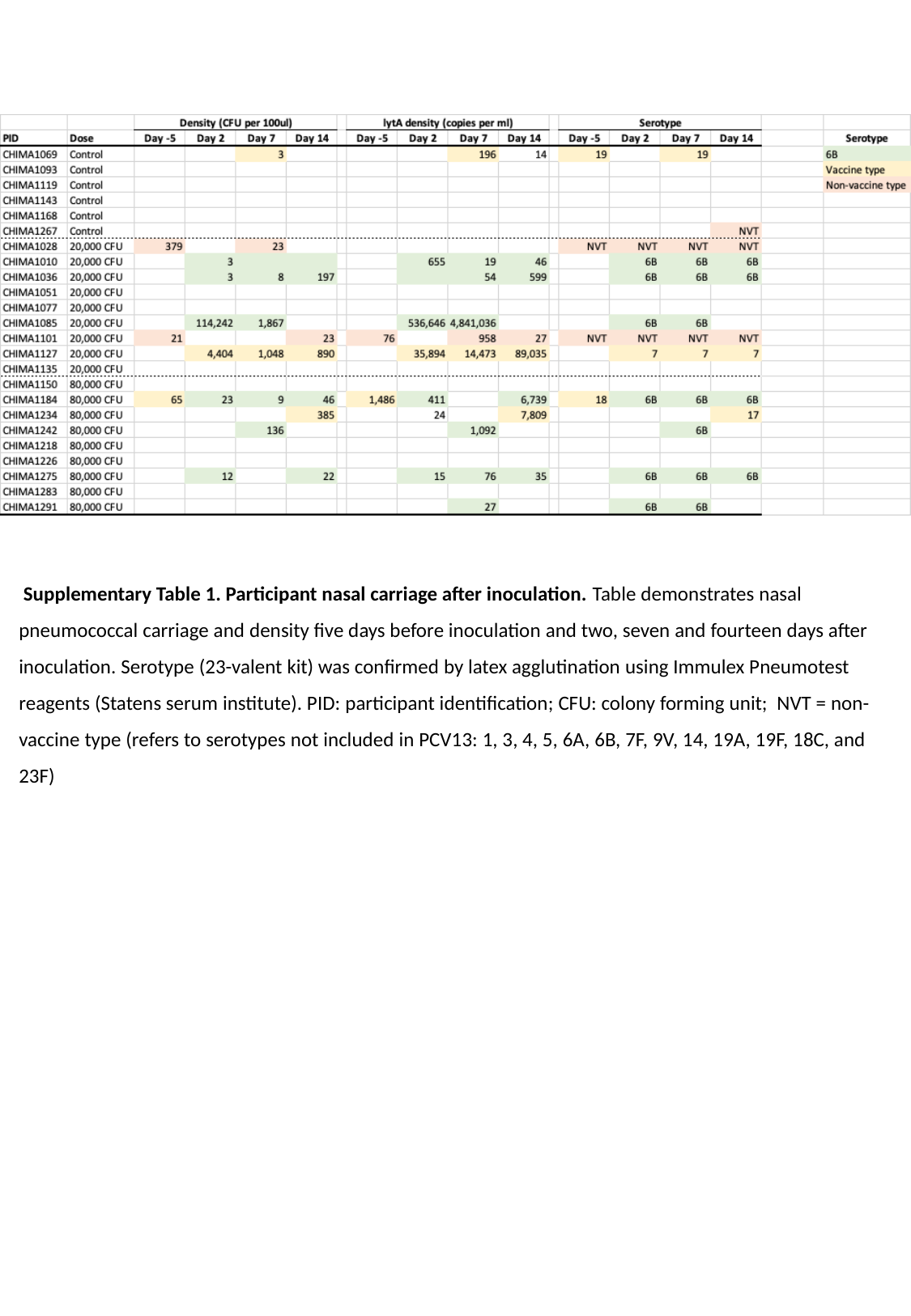

Supplementary Table 1. Participant nasal carriage after inoculation. Table demonstrates nasal pneumococcal carriage and density five days before inoculation and two, seven and fourteen days after inoculation. Serotype (23-valent kit) was confirmed by latex agglutination using Immulex Pneumotest reagents (Statens serum institute). PID: participant identification; CFU: colony forming unit; NVT = non-vaccine type (refers to serotypes not included in PCV13: 1, 3, 4, 5, 6A, 6B, 7F, 9V, 14, 19A, 19F, 18C, and 23F)

## Slide 2
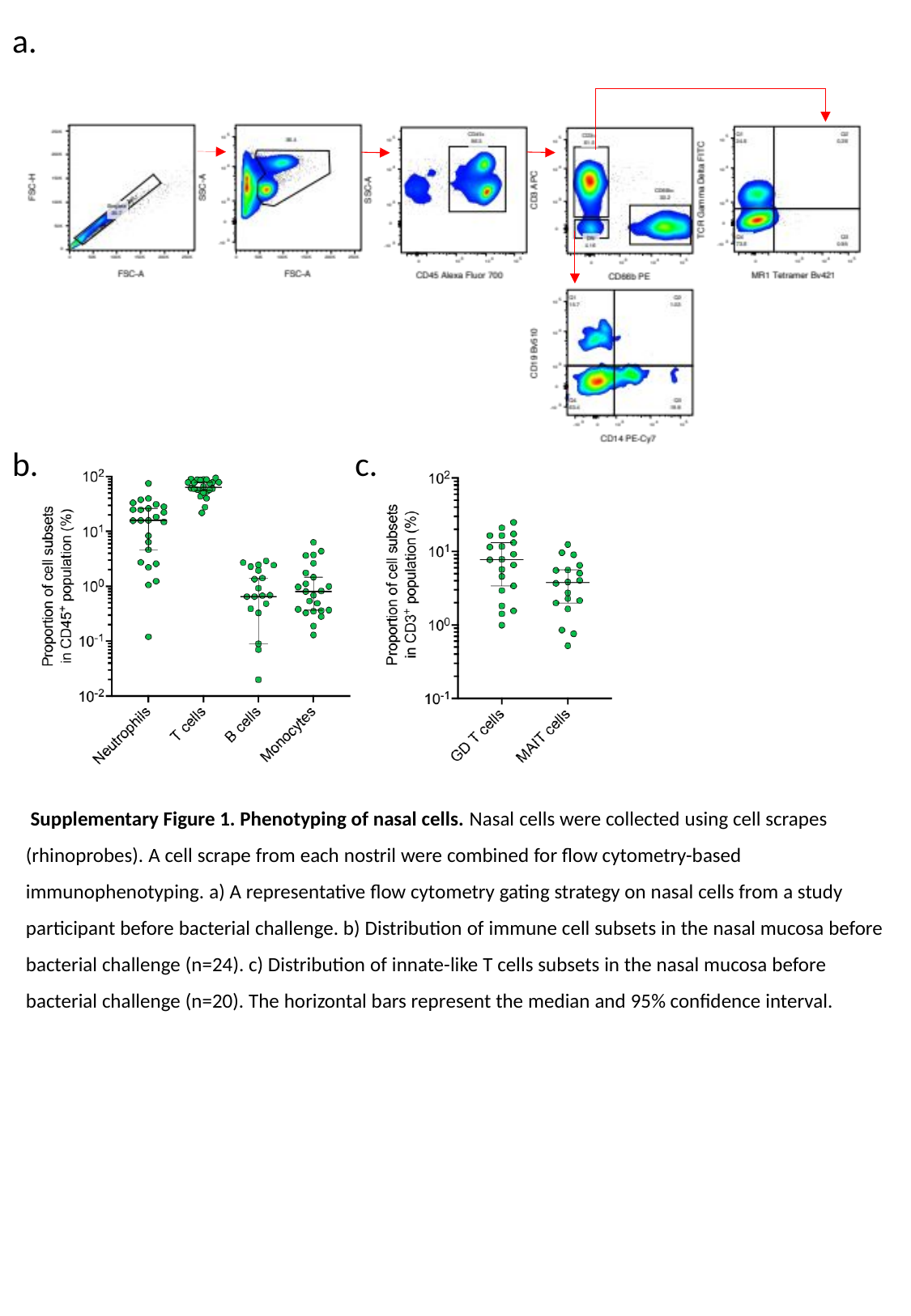

a.
b.
c.
 Supplementary Figure 1. Phenotyping of nasal cells. Nasal cells were collected using cell scrapes (rhinoprobes). A cell scrape from each nostril were combined for flow cytometry-based immunophenotyping. a) A representative flow cytometry gating strategy on nasal cells from a study participant before bacterial challenge. b) Distribution of immune cell subsets in the nasal mucosa before bacterial challenge (n=24). c) Distribution of innate-like T cells subsets in the nasal mucosa before bacterial challenge (n=20). The horizontal bars represent the median and 95% confidence interval.

## Slide 3
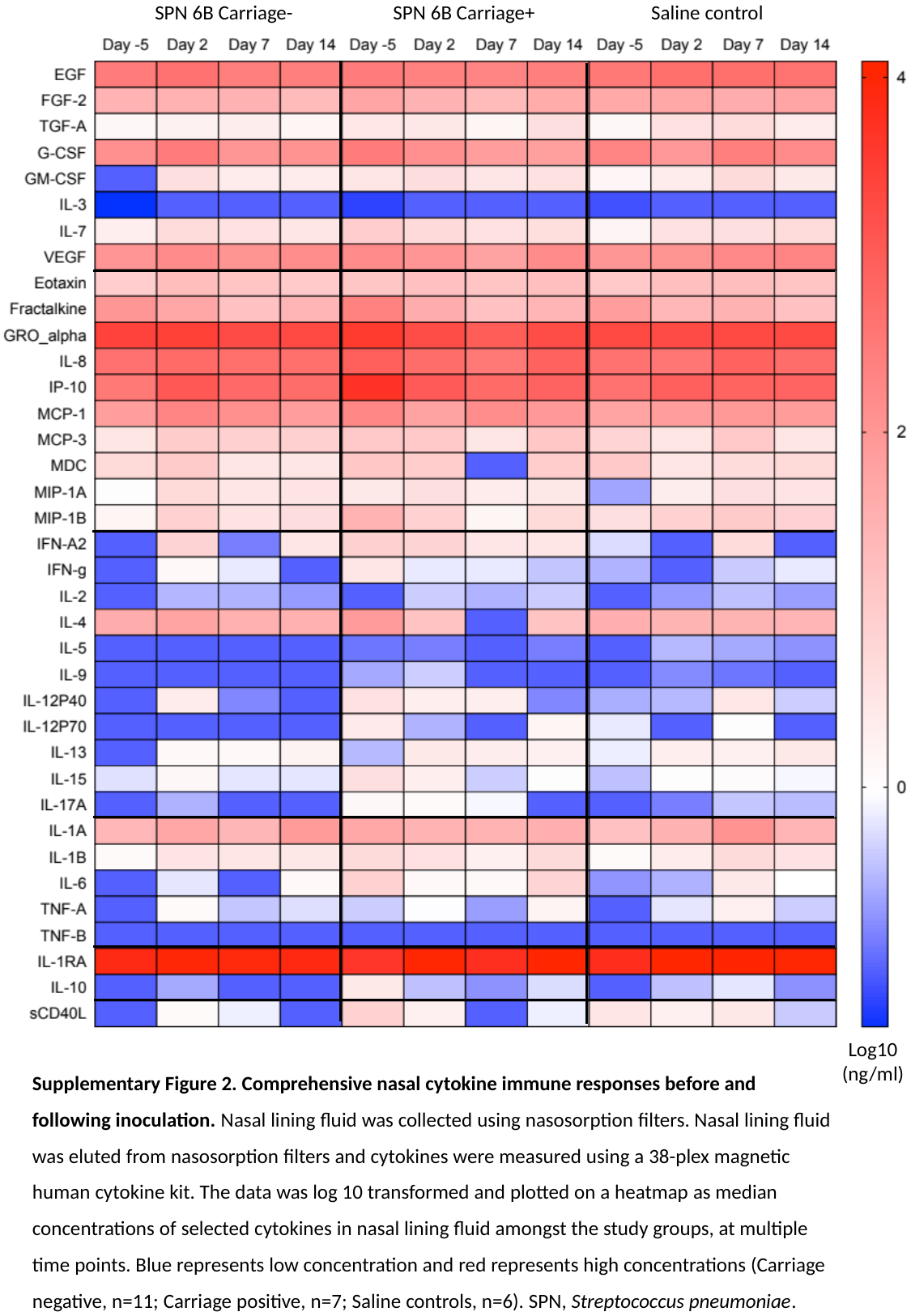

SPN 6B Carriage-
SPN 6B Carriage+
Saline control
Log10
(ng/ml)
Supplementary Figure 2. Comprehensive nasal cytokine immune responses before and following inoculation. Nasal lining fluid was collected using nasosorption filters. Nasal lining fluid was eluted from nasosorption filters and cytokines were measured using a 38-plex magnetic human cytokine kit. The data was log 10 transformed and plotted on a heatmap as median concentrations of selected cytokines in nasal lining fluid amongst the study groups, at multiple time points. Blue represents low concentration and red represents high concentrations (Carriage negative, n=11; Carriage positive, n=7; Saline controls, n=6). SPN, Streptococcus pneumoniae.

## Slide 4
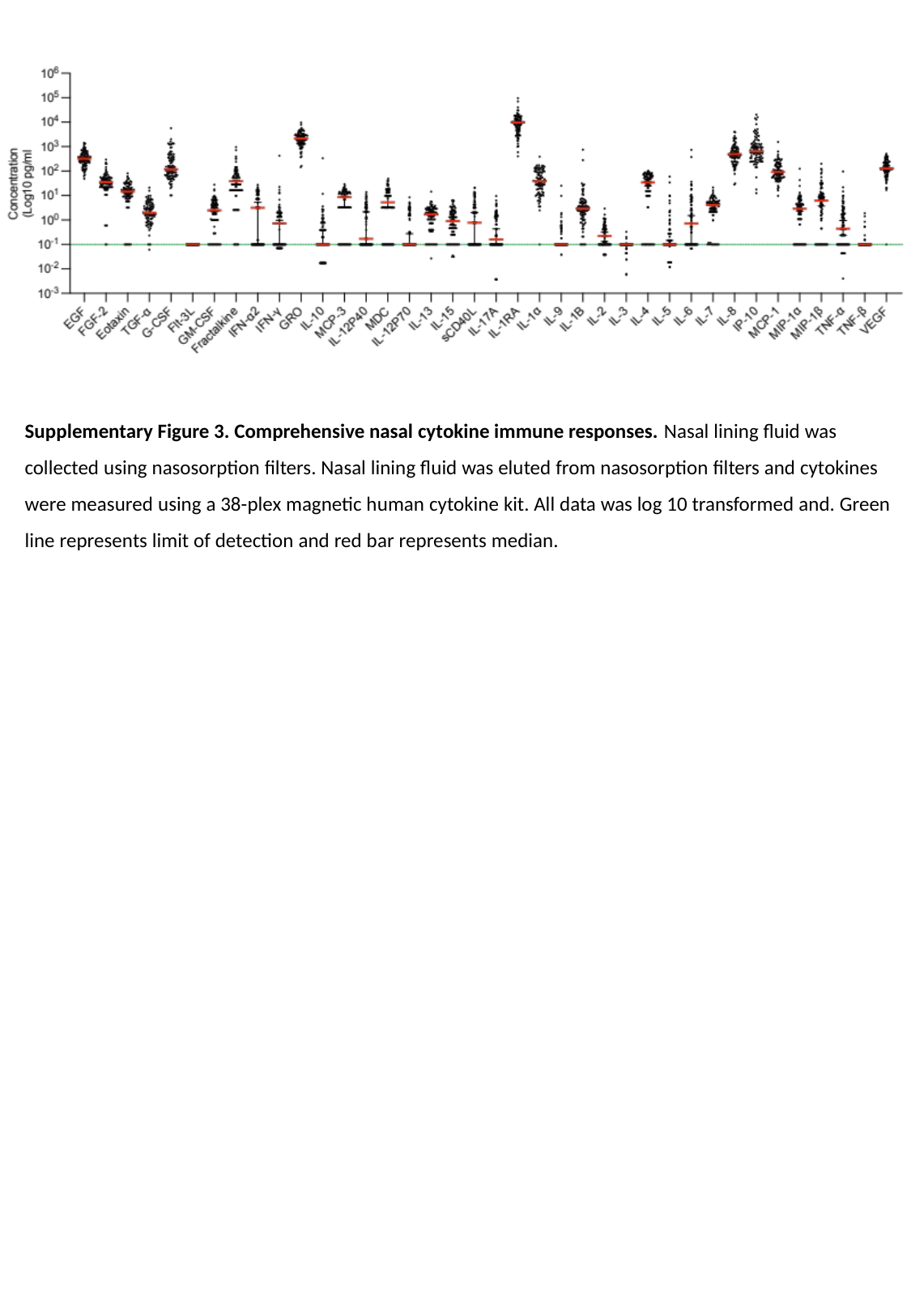

Supplementary Figure 3. Comprehensive nasal cytokine immune responses. Nasal lining fluid was collected using nasosorption filters. Nasal lining fluid was eluted from nasosorption filters and cytokines were measured using a 38-plex magnetic human cytokine kit. All data was log 10 transformed and. Green line represents limit of detection and red bar represents median.
