# Supplementary material for: A feasibility study of controlled human infection with Streptococcus pneumoniae in Malawi
Source: eBioMedicine. 2021 Sep 24;72:103579. doi: 10.1016/j.ebiom.2021.103579 (PMC8479630; doi:10.1016/j.ebiom.2021.103579)
Supplement: Supplementary file 2 [file mmc2.docx]

MARVELS Consortium Supplemental File

| **First names (will be abbreviated on Pubmed)** | **Surnames (not abbreviated)** |
| --- | --- |
| Clara | Ngoliwa |
| Edward | Mangani |
| Modesta | Reuben |
| Vitumbiku | Nkhoma |
| Helen | Thomson |
| Christopher | Mkandawire |
| Simon | Sichone |
| Raphael | Kamng’ona |
| Mphatso | Mayuni |
| Percy | Mwenechanya |
| Asia | Sophia-Wolf |
| Godwin | Tembo |
| Bridgette | Galafa |
| Neema | Toto |
| Tina | Harawa |
| Blessings | Kapumba |
| Joel | Gondwe |
| Clemens | Masesa |
| Sandra | Antoine |
| Kate | Gooding |
| Markus | Gmeiner |
| Mike | Parker |
| Andrew | Pollard |
| Rob | Heyderman |
| Jason | Hinds |
| Mark | Alderson |
| Chris | Bailey |
| Marien | de Jonge |
| Robert | Kneller |
| Jeremy | Brown |
| Jane | Mallewa |
| David | Goldblatt |
| Richard | Malley |
| Jeff | Weiser |
| Jonathon | Grigg |
| Henry | Mwandumba |
| Debby | Bogaert |
