# Supplementary material for: A feasibility study of controlled human infection with Streptococcus pneumoniae in Malawi
Source: eBioMedicine. 2021 Sep 24;72:103579. doi: 10.1016/j.ebiom.2021.103579 (PMC8479630; doi:10.1016/j.ebiom.2021.103579)
Supplement: Supplementary file 4 [file mmc4.docx]

# Caption for supplementary material

## CONSORT checklist

Document signposts required reporting information for this feasibility study

## MARVELS Consortium Supplemental File

Document provides first names and surnames for members of the MARVELS Consortium

## Antibody list

File describing the flow cytometry antibodies used for this study

## Culture vs. lytA PCR

Data file describing the relationship between classical microbiological and molecular testing of nasal wash for pneumococcus

## Supplementary Figures

### Supplementary Table 1

Details individualised nasal carriage of pneumococcus for each study participant

### Supplementary Figure 1

Details cellular phenotyping data from nasal scrape samples

### Supplementary Figure 2

Heat map of nasal cytokine data from nasosorption samples

### Supplementary Figure 3

Details comprehensive nasal cytokine data from nasosorption samples used to construct Figure 4 and Supplementary Figure 2.
